# The chemistry of the nitrate–nitrite–nitric oxide pathway: regulating muscle oxygenation and exercise performance

**DOI:** 10.1039/d6ra00317f

**Published:** 2026-03-20

**Authors:** Jing Liang, Taibin Huang, Jinping Li, Zhiyu Yang, Jin Ni, Yanchao Wang

**Affiliations:** a School of Physical Education, Xinyu University Xinyu Jiangxi 338004 China liangxiaping@163.com

## Abstract

Nitric oxide (NO) is a pleiotropic signaling molecule fundamentally involved in regulating skeletal muscle physiology, including blood flow, contractility, and metabolism. For decades, the synthesis of NO was attributed solely to the l-arginine-dependent nitric oxide synthase (NOS) enzymes. However, the discovery and characterization of the nitrate–nitrite–NO pathway have revealed an alternative, NOS-independent mechanism for NO generation. This pathway is particularly significant under hypoxic and acidic conditions, which are characteristic of exercising skeletal muscle. Dietary inorganic nitrate, abundant in green leafy vegetables and beetroot, is sequentially reduced to nitrite and then to bioactive NO. This review critically examines the intricate chemistry underpinning this pathway, from the initial enzymatic reduction of nitrate by both mammalian and microbial reductases to the diverse chemical routes of nitrite reduction to NO within the muscle milieu. We delve into the specific roles of key proteins such as xanthine oxidoreductase, deoxyhemoglobin/deoxymyoglobin, and mitochondrial complexes in catalyzing these transformations. Furthermore, we explore how NO generated *via* this pathway modulates muscle oxygenation through vasodilation and regulation of mitochondrial respiration. The ergogenic potential of dietary nitrate supplementation is discussed in the context of human exercise performance, highlighting the significant controversies, methodological challenges, and sources of inter-individual variability, including genetics and the microbiome. This review aims to provide a comprehensive, chemistry-focused perspective on the nitrate–nitrite–NO pathway, bridging fundamental biochemical mechanisms with their physiological consequences in exercise.

## Abstract 2

This review comprehensively elucidates the redox chemistry and physiological integration of the nitrate–nitrite–nitric oxide pathway in regulating skeletal muscle oxygenation and exercise performance.

## Graphical abstract

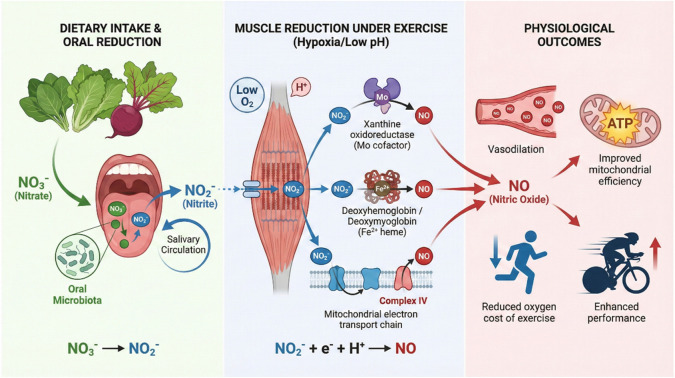

## 1. Introduction

**Fig. 1:**
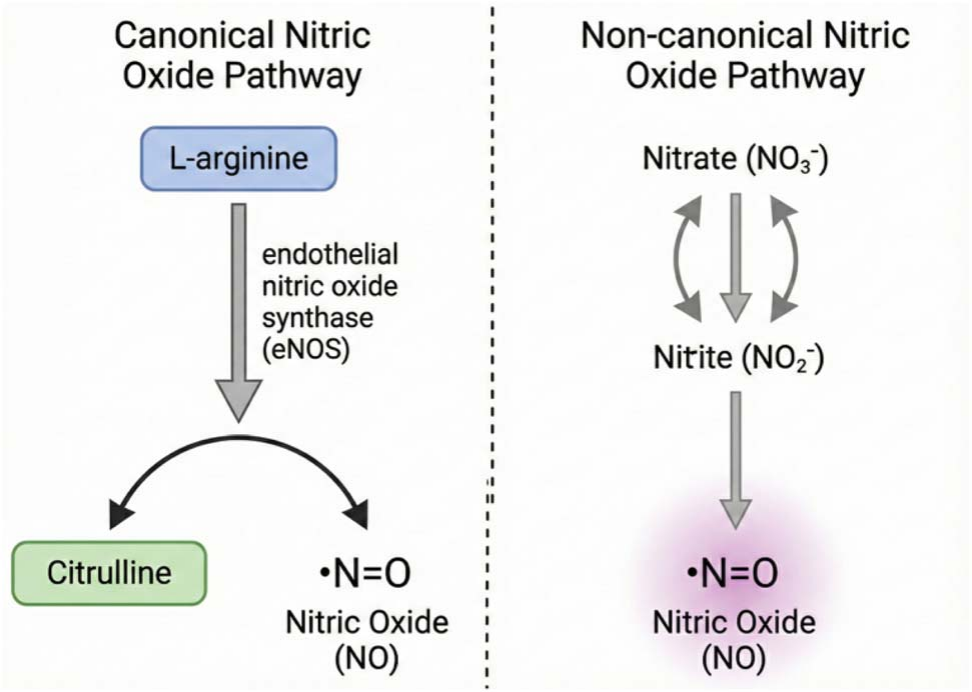
Overview of NO generation pathways. Schematic representation of the canonical l-arginine-NOS pathway and the non-canonical nitrate–nitrite–NO pathway.

Nitric oxide (NO), a simple diatomic molecule, functions as a critical signaling gasotransmitter in mammalian physiology, orchestrating a vast array of biological processes.^1^ Within the context of skeletal muscle, its influence is profound, impacting vasoregulation, excitation-contraction coupling, glucose uptake, and mitochondrial bioenergetics.^2,3^ The canonical pathway for NO synthesis involves a family of nitric oxide synthase (NOS) enzymes—neuronal (nNOS), inducible (iNOS), and endothelial (eNOS)—which catalyze the five-electron oxidation of l-arginine to l-citrulline and NO^4–6^ (Fig. 1). This reaction is oxygen-dependent, with its efficiency markedly decreasing as oxygen tension falls.^7^ This oxygen dependency presents a physiological paradox, as the demand for NO-mediated vasodilation and metabolic regulation is often greatest during periods of intense physical exercise when muscle oxygen availability (PO_2_) can plummet to hypoxic or even anoxic levels.^8^ This paradox is resolved by the existence of a complementary, NOS-independent pathway for NO generation: the nitrate–nitrite–NO pathway.^9^ This pathway utilizes inorganic nitrate (NO_3_^−^), derived primarily from dietary sources such as beetroot and leafy green vegetables, as a substrate for NO synthesis.^10^ The process begins with the reduction of relatively inert nitrate to the more reactive intermediate, nitrite (NO_2_^−^).^11^ This initial step is primarily accomplished by commensal anaerobic bacteria in the oral cavity and gastrointestinal tract, which possess potent nitrate reductase enzymes.^12^ Absorbed nitrite, along with endogenously formed nitrite, circulates in the blood and can be further reduced to NO. Crucially, this second reduction step is markedly enhanced by low pH and hypoxia, conditions that prevail in contracting skeletal muscle, thereby elegantly coupling NO production to metabolic demand at the precise time and location it is needed.^12,13^

The growing appreciation for this pathway has catalyzed a surge of research into the ergogenic potential of dietary nitrate supplementation.^14^ Numerous studies have reported that augmenting nitrate intake can reduce the oxygen cost of submaximal exercise, improve mitochondrial efficiency, and enhance performance in a range of exercise modalities.^15,16^ However, the field is also characterized by significant controversy and conflicting results, particularly concerning the efficacy of supplementation in elite athletes and the optimal dosing strategies.^17–19^ Furthermore, substantial inter-individual variability in response poses a major challenge to its widespread application.^20^ This review contends that a deeper understanding of the fundamental chemistry and biochemistry of the nitrate–nitrite–NO pathway is essential to resolve these controversies and unlock its full potential. Therefore, the primary objective of this paper is to provide a comprehensive and critical analysis of the chemical mechanisms governing each step of this pathway within the skeletal muscle environment. We will dissect the kinetics and redox chemistry of nitrate and nitrite reduction by key mammalian enzymes and proteins. We will then connect this molecular chemistry to the physiological regulation of muscle oxygenation and analyze the contentious evidence regarding exercise performance enhancement. Finally, we will address the significant methodological hurdles in measuring NO bioavailability and outline future research directions required to advance the field. In doing so, this review aims to serve as a key resource for chemists, biochemists, and physiologists interested in the intricate interplay between inorganic anions, redox biology, and human physical performance.

## 2. Chemical basis of the nitrate–nitrite–nitric oxide pathway

The transformation of inorganic nitrate into bioactive nitric oxide is a multi-step redox process involving a fascinating interplay between host and microbial enzymes, as well as non-enzymatic chemical reactions driven by the unique biochemical environment of exercising tissue.^21^ This section deconstructs the chemical reactions and molecular players that define this pathway.

### 2.1. The reduction of nitrate to nitrite

The initial and often rate-limiting step in this pathway is the two-electron reduction of nitrate to nitrite (eqn (1)). The standard reduction potential (*E*°′) for the NO_3_^−^/NO_2_^−^ couple is +0.42 V, making it thermodynamically feasible for various biological reductants to drive this reaction.^22^

1 NO_3_^−^ + 2e^−^ + 2H^+^ → NO_2_^−^ + H_2_O

While once thought to be metabolically inert in mammals, it is now clear that nitrate can be reduced endogenously, although the primary contribution under normal conditions comes from external microbial sources.

Microbial nitrate reduction: the vast majority of dietary nitrate reduction occurs *via* the entero-salivary circulation.^23^ Following ingestion, nitrate is rapidly absorbed in the upper gastrointestinal tract, leading to a peak in plasma concentration after 1–2 hours.^12^ Approximately 25% of this circulating nitrate is actively taken up by the salivary glands and concentrated in saliva to levels 10–20 times higher than in plasma.^24^ The primary site for microbial nitrate reduction is the oral cavity, specifically the deep clefts on the dorsal surface of the tongue. Here, commensal facultative anaerobic bacteria—predominantly from the genera *Veillonella*, *Actinomyces*, and *Rothia*—utilize nitrate as an alternative electron acceptor for respiration *via* powerful nitrate reductases.^12,25^ Following this initial oral reduction, swallowed nitrite enters the acidic environment of the stomach. While some is protonated and reduced to NO locally, the remainder is absorbed into the systemic circulation. Furthermore, while the oral cavity is the primary site, the lower gastrointestinal tract also harbors a diverse microbiome capable of nitrate and nitrite reduction, contributing to the systemic pool of these metabolites.^12,26^ The critical role of this oral microbial step has been demonstrated in studies where the use of antibacterial mouthwash abolishes the rise in plasma nitrite and negates the ergogenic effects of nitrate supplementation.^27,28^

Mammalian nitrate reduction: while less efficient than microbial reduction, evidence for endogenous mammalian nitrate reductase activity is now compelling.^29^ Skeletal muscle, which serves as a significant storage reservoir for nitrate,^30^ possesses the enzymatic machinery to reduce it to nitrite.^31^ The primary enzyme implicated is xanthine oxidoreductase (XOR), a complex molybdo–flavoprotein. XOR is a versatile enzyme that exists in two interconvertible forms: xanthine dehydrogenase (XDH), which preferentially uses NAD^+^ as an electron acceptor, and xanthine oxidase (XO), which uses O_2_.^14,32,33^ Under ischemic or hypoxic conditions, the enzyme shifts towards the XO form and can utilize its molybdenum cofactor (Moco) to transfer electrons to alternative substrates, including nitrate and nitrite. The reaction involves the Mo center cycling between the Mo(vi) and Mo(iv) oxidation states, with electrons supplied by physiological reductants (*e.g.*, NADH) or purine substrates (xanthine/hypoxanthine) *via* electron flow through the FAD and iron–sulfur centers. To make the quantitative constraints of this pathway explicit, Table 1 summarizes representative kinetic parameters reported for purified XOR/XO systems, including substrate/electron-donor affinities, competitive effects of purines, and characteristic intramolecular electron-transfer rates that set an upper bound on catalytic throughput.^34^ These values are strongly modulated by pH and oxygen tension, and they should be interpreted as mechanistic benchmarks rather than direct measurements in intact human muscle preparations.^35^

**Table 1:** Representative kinetic and electron transfer parameters of xanthine oxidoreductase relevant to nitrate and nitrite reduction under hypoxic conditions

Process class	Parameter (what it represents)	Representative value (s)	Typical conditions noted in source	Primary source (s)
Binding of substrates/cofactors to XOR	Km (NO_3_^−^) for XOR-driven nitrate reduction	∼32 mM (inorganic nitrate, NaNO_3_)	Purified XO/XOR; kinetics compiled across studies	36
	Km (NO_2_^−^) at Mo-cofactor for XOR nitrite reduction to NO	Commonly reported ∼ mM range; review notes ∼2.5 mM often used/observed in XO systems	Anoxic/hypoxic conditions emphasized; pH dependence noted	
	Km (xanthine) at Mo-cofactor (electron donor substrate)	6.5 µM	Purified XO; anoxic kinetics discussion	
	Km (NADH) at FAD site (electron donor)	121.7 µM (XO form); 6.7 µM (XDH form)	Highlights form-dependent affinity differences	
	Ki (xanthine) inhibiting NO_2_^−^reduction (competition at Mo-cofactor)	55 µM	Anoxic XO nitrite-reduction context	
	Km range for organic nitrate (GTN) reduced by XOR	330–500 µM	Reported as comparison to inorganic nitrate kinetic hurdle	
Rate of electron transfer (intramolecular)	Mo ↔ Fe/S electron equilibration (first-order)	∼8.5 × 10^3^ s^−1^	Described as particularly rapid internal ET within XO	37 and 38
	Coupled ET step exhibiting pH dependence (example benchmark)	∼155 s^−1^ (pH 6) to ∼330 s^−1^ (pH 10)	Reported as modest linear pH dependence in complex flavoproteins (context includes XO)	
Reduction chemistry	Overall stoichiometry for nitrate reduction (as framed in manuscript)	NO_3_^−^ + 2e^−^ + 2H^+^ → NO_2_^−^ + H_2_O	Reaction identity; kinetics depend on electron donor, pH, O2	38

### 2.2. The reduction of nitrite to nitric oxide

The conversion of nitrite to NO is the key bioactive step, involving a one-electron reduction (eqn (2)). This process is facilitated by a remarkable variety of chemical and biochemical mechanisms that are potently activated by the physiological stresses of exercise.

2 NO_2_^−^ + e^−^ + H^+^ → NO + OH^−^

The standard reduction potential (*E*°′) for the NO_2_^−^/NO couple is approximately +0.37 V at pH 7, but it becomes much more favorable as the pH drops (*e.g.*, ∼+0.6 V at pH 6), providing a thermodynamic basis for its enhancement in acidic environments.^22^

Non-enzymatic acidic disproportionation: in an acidic solution, nitrite can undergo a disproportionation reaction. Two molecules of nitrous acid (HNO_2_), the protonated form of nitrite (pKa ∼3.4), react to form dinitrogen trioxide (N_2_O_3_), which then decomposes into NO and nitrogen dioxide (NO_2_).^39^

3 2HNO_2_ ⇌ N_2_O_3_ + H_2_O → NO + NO_2_ + H_2_O

While the pH of exercising muscle rarely drops below 6.5, this chemical pathway may still contribute to NO generation in localized acidic microdomains, for instance near the mitochondrial membrane or within endosomes.^40^ The kinetics of this non-enzymatic NO generation can be described by the rate equation: *d*_[NO]_/*d*_t_ = *k*_obs_[NO_2_^−^]^2^[H^+^]^2^. The observed rate constant (*k*_obs_) is highly pH-dependent. While the reaction is rapid at gastric pH (<3.0), at the physiological pH of exercising muscle (typically 6.0–6.8), the bulk rate is relatively slow. However, because the rate increases 10-fold for every one-unit decrease in pH (due to the squared proton term), this pathway becomes mathematically and physiologically significant in highly acidic localized microdomains, such as near the mitochondrial inner membrane.^12^

Deoxyhemoglobin and deoxymyoglobin: perhaps the most physiologically elegant mechanism for hypoxic NO generation involves the ferrous (Fe^2+^) heme proteins, deoxyhemoglobin in red blood cells and deoxymyoglobin in muscle cells.^41,42^ These proteins act as allosterically controlled nitrite reductases. When oxygenated, the heme iron is sterically hindered and largely unreactive with nitrite. However, upon deoxygenation, a conformational change exposes the heme iron, allowing it to bind and reduce nitrite in a one-electron transfer, producing NO and ferric (Fe^3+^) methemoglobin/metmyoglobin.^43^

4 Hb(Fe^2+^) + NO_2_^−^ + H^+^ → Hb(Fe^3+^) + NO + OH^−^

**Fig. 2:**
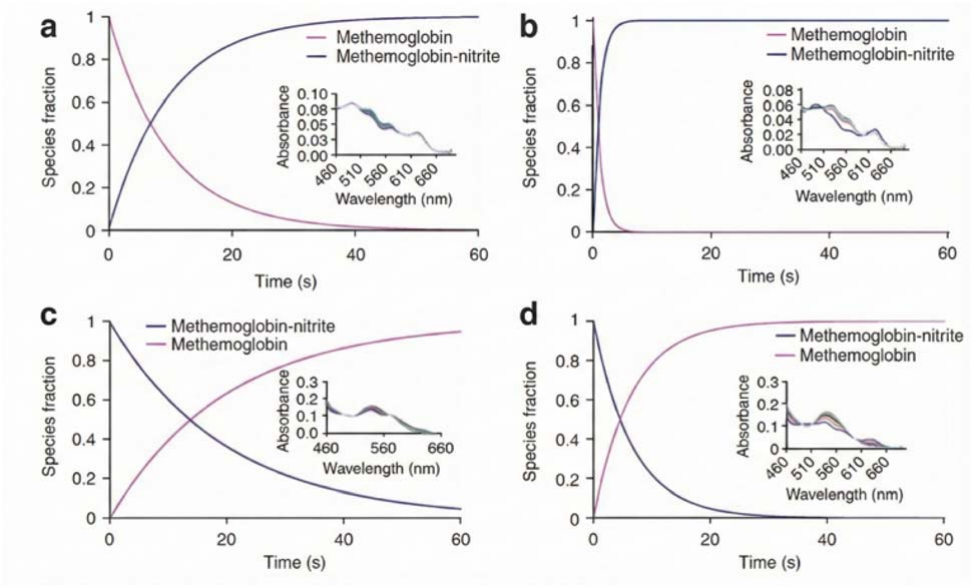
Kinetics of nitrite binding and dissociation from methemoglobin as a function of pH. (a) The kinetics of nitrite (4 mM) association with MetHb (50 mM) at pH 7.4 using stopped-flow absorption spectroscopy. The observed rate of nitrite binding averaged 0.12 ± 0.01 s^−1^ (*n* ¼ 6). Inset depicts raw data. (b) The kinetics of nitrite (4 mM) association with MetHb (50 mM) at pH 6. The observed rate of nitrite binding averaged 0.76 ± 0.11 s^−1^ (*n* ¼ 6). Inset depicts raw data. (c) The kinetics of nitrite dissociation from MetHb for the reaction of 2.5 mM HbFe(iii)–NO_2_– with 2.5 mM KCN at pH 7.4. The rate of nitrite dissociation averaged 0.053 ± 0.002 s–1 (*n* ¼ 3). Inset depicts raw data. (d) The kinetics of nitrite dissociation from MetHb for the reaction of 2.5 mM HbFe(iii)–NO_2_– with 2.5 mM KCN at pH 6. The rate of nitrite dissociation averaged 0.153 ± 0.001 s–1 (*n* ¼ 3).^53^

Fig. 2 shows the kinetics of nitrite binding and dissociation from methemoglobin as a function of pH. The bell-shaped oxygen dependence can be expressed explicitly by coupling oxygen tension (*P*_O_2__) to the concentration of available ferrous deoxy-hemes and the allosteric dependence of the nitrite-reduction rate constant on hemoglobin quaternary state. First, hemoglobin oxygen fractional saturation (*Y*) may be approximated by a Hill form, *Y*(*P*_O_2__} = *P*_O_2__^*n*^/(*P*_50_^*n*^ + *P*_O_2__^*n*^), giving the concentration of vacant deoxy-hemes as [HbFe(ii)]_deoxy_ ≈ [Hb]_heme_(1 − *Y*). Second, the apparent bimolecular rate constant for nitrite reduction is state-weighted, so that *k*_app_}(*P*_O_2__) = *k*_R_·*f*_R_(*P*_O_2__) + *k*_T_·*f*_T_(*P*_O_2__) with *f*_R_ + *f*_T_ = 1 (*R*-state character rises as O_2_ ligation increases, whereas substrate availability [HbFe(ii)]_deoxy_ falls).^43,44^ Under pseudo-first-order nitrite conditions, the initial NO-generation rate can therefore be written as *v*_NO_(*P*_O_2__) ≈ *k*_app_(*P*_O_2__)·[HbFe(ii)]_deoxy_(*P*_O_2__)·[NO_2_^−^]·[H^+^], making the origin of the bell-shaped dependence transparent: increasing *P*_O_2__ raises *k*_app_ (more *R*-state) but reduces [HbFe(ii)]_deoxy_ (less substrate), yielding a maximum near *P*_50_.^45^ Representative parameters reported at 37 °C place *k*_R_ (*R*-state HbA_0_) ≈ 12 M^−1^ s^−1^ and *k*_T_ (T-state HbA_0_) ≈ 0.2 M^−1^ s^−1^ (∼60-fold difference), while myoglobin nitrite-reductase kinetics are of similar order (∼12 M^−1^ s^−1^), consistent with enhanced nitrite-to-NO conversion under partial deoxygenation around *P*_50_.^46^

**Fig. 3:**
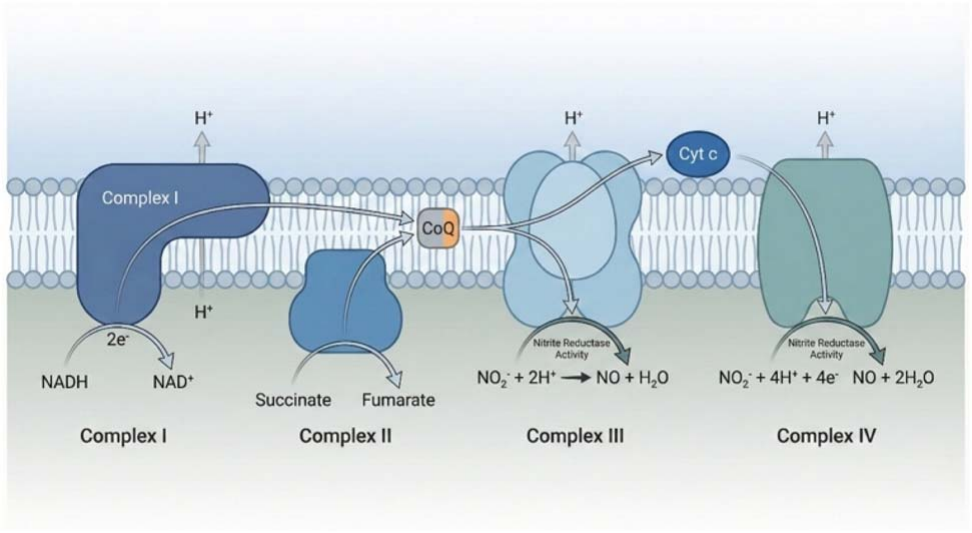
Nitrite reduction within the mitochondrial ETC.

Mitochondrial nitrite reduction: mitochondria, the cell's metabolic powerhouses, have emerged as another key site for nitrite reduction to NO, particularly under hypoxic conditions. Several components of the electron transport chain (ETC) can function as nitrite reductases (Fig. 3). Cytochrome c oxidase (CcO, Complex IV), the terminal enzyme of the ETC, can reduce nitrite to NO when its oxygen-binding site is vacant due to hypoxia.^46,47^ This reaction is competitively inhibited by oxygen, ensuring it only occurs when cellular PO_2_ is very low.^12,48^ This mitochondrial NO production can then act locally to modulate respiration itself, creating an important autoregulatory loop.^49^ Other ETC components, including Complex I and Complex III, have also been shown to contribute to nitrite reduction, expanding the capacity of mitochondria to act as cellular hubs for NO signaling under hypoxia.^50^ While the detailed molecular mechanisms are still under investigation, and specific kinetic parameters like activation energies for human muscle mitochondria have not been determined, this pathway represents a direct link between cellular energy status and NO bioavailability.^51,52^

Other enzymatic pathways: in addition to the major players described above, other enzymes can contribute to nitrite reduction. XOR, which reduces nitrate, can also further reduce nitrite to NO at its molybdenum site, especially under acidic conditions.^54^ Carbonic anhydrase (CA) does not directly reduce nitrite, as its active site zinc ion is not redox-active. Instead, CA acts as a ‘nitrous anhydrase,’ facilitating the reaction of nitrite with protons to form nitrous acid (HNO_2_), and subsequently catalyzing the dehydration of HNO_2_ to form dinitrogen trioxide (N_2_O_3_). The N_2_O_3_ intermediate then spontaneously homolyzes to yield NO and NO_2_. Thus, CA accelerates NO generation *via* a disproportionation mechanism rather than a direct enzymatic reduction.^55^ The collective action of these diverse pathways ensures a robust and redundant system for generating NO from the nitrate–nitrite pool, finely tuned to the metabolic state of the muscle tissue.^56,57^

To consolidate the mechanistic diversity described above, Table 2 integrates the key enzymes and proteins involved in nitrate and nitrite reduction with their cellular localization, activating conditions, and defining chemical cofactors. This comparison highlights a unifying redox principle: nitrate- and nitrite-derived NO generation is preferentially enabled under hypoxia and acidosis, in contrast to the oxygen-dependent constraints of classical NOS catalysis. Importantly, the convergence of molybdenum-based (XOR), heme-based (deoxyHb/Mb), and mitochondrial metal centers underscores the chemical redundancy built into this pathway, ensuring NO bioavailability across spatial and metabolic gradients within muscle.^58,59^

**Table 2:** Key enzymes and proteins in the nitrate–nitrite–NO pathway

Enzyme/protein	Location	Reaction catalyzed	Activating conditions	Key chemical feature	References
Bacterial nitrate reductase	Oral cavity, gut	NO_3_^−^ → NO_2_^−^	Anaerobiosis	Molybdenum or tungsten cofactor	12 and 26
XOR	Skeletal muscle, liver, endothelium	NO_3_^−^ → NO_2_^−^; NO_2_^−^ → NO	Hypoxia, acidosis	Molybdenum cofactor, FAD, Fe–S clusters	32, 34 and 60
Deoxyhemoglobin/Deoxymyoglobin	Red blood cells/Muscle cells	NO_2_^−^ → NO	Hypoxia (low O_2_ saturation)	Ferrous (Fe^2+^) heme iron	42 and 45
Cytochrome c oxidase	Inner mitochondrial membrane	NO_2_^−^ → NO	Severe hypoxia	Heme a_3_-CuB binuclear center	46 and 49
Carbonic anhydrase	Red blood cells, endothelium	Accelerates HNO_2_ decomposition	Acidosis	Zinc-coordinated hydroxide ion	55 and 61

## 3. Regulation of muscle oxygenation *via* the nitrate–nitrite–NO pathway

The NO produced from the nitrate–nitrite pathway exerts profound effects on skeletal muscle physiology, primarily by influencing the dynamics of oxygen supply and demand. This regulation occurs through two principal mechanisms: enhancing oxygen delivery *via* vasodilation and modulating oxygen consumption at the mitochondrial level.

### 3.1. NO-mediated vasodilation and muscle blood flow

A cornerstone of NO biology is its role as a potent vasodilator. NO generated in or near the vascular smooth muscle cells diffuses to its intracellular receptor, soluble guanylate cyclase (sGC).^62,63^ The binding of NO to the ferrous heme moiety of sGC triggers a conformational change that activates the enzyme, leading to the conversion of guanosine triphosphate (GTP) to cyclic guanosine monophosphate (cGMP).^6,64^

**Fig. 4:**
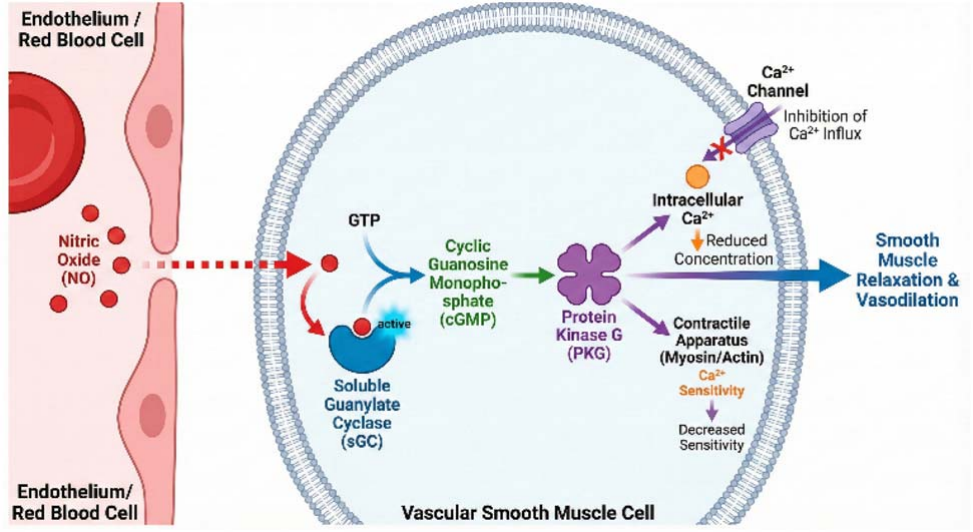
NO/sGC/cGMP signaling pathway in vascular smooth muscle.

This elevation in cGMP activates protein kinase G (PKG), which phosphorylates several downstream targets that collectively reduce intracellular calcium concentrations and desensitize the contractile apparatus to calcium, resulting in smooth muscle relaxation and vasodilation (Fig. 4).^65^ By increasing the diameter of arterioles feeding the muscle bed, this signaling cascade reduces vascular resistance and increases blood flow, a phenomenon known as functional hyperemia.

The nitrate–nitrite–NO pathway is particularly well-suited to contribute to exercise-induced functional hyperemia. As muscle contracts, local PO_2_ falls and pH decreases due to lactic acid and CO_2_ production. These are the precise chemical triggers that activate nitrite reduction by deoxyhemoglobin, deoxymyoglobin, and XOR.^42,66^ This ensures that NO production is spatially and temporally matched to the metabolic needs of the active muscle fibers, directing blood flow preferentially to areas with the highest oxygen demand.^67,68^ For example, studies using intra-arterial nitrite infusions in humans have demonstrated vasodilation and increased forearm blood flow, an effect that is more pronounced during exercise, confirming the physiological relevance of this mechanism.^69^

### 3.2. Modulation of mitochondrial respiration and metabolic efficiency

Beyond its vascular effects, NO directly interacts with the machinery of cellular respiration within the mitochondria. The primary target is Cytochrome c Oxidase (CcO, Complex IV), the terminal enzyme of the ETC.^42^ NO can bind reversibly to the reduced ferrous heme (*a*_3_^2+^) in the binuclear center of CcO, competing directly with oxygen for this binding site.^42^ This reversible inhibition slows the rate of electron transport and, consequently, reduces mitochondrial oxygen consumption (VO_2_).

The physiological consequence of this interaction is complex and appears to be concentration-dependent.^70^ At very high, pathophysiological concentrations, NO can cause potent and sustained inhibition of respiration, leading to cellular damage.^71,72^ However, at lower, physiological concentrations, such as those likely produced by the nitrate–nitrite pathway during exercise, this inhibitory effect may be beneficial. By transiently “throttling” respiration, NO may improve the overall efficiency of oxidative phosphorylation.^73^ This concept, often termed “oxygen sparing,” suggests that for a given amount of ATP produced, less oxygen is consumed. The proposed mechanism involves a reduction in proton leak across the inner mitochondrial membrane, which improves the coupling of electron transport to ATP synthesis (*i.e.*, increases the P/O ratio).^42^

It is crucial to note, however, that the nitrate–nitrite–NO pathway must be contextualized within the broader redox environment of the exercising muscle. During intense exercise, elevated oxidative stress leads to the production of superoxide (O_2_˙^−^). Nitric oxide rapidly reacts with superoxide at a diffusion-limited rate to form peroxynitrite (ONOO^−^), a highly reactive nitrogen species (RNS). While physiological NO levels transiently and reversibly inhibit cytochrome c oxidase, the formation of peroxynitrite under conditions of oxidative stress can lead to irreversible inhibition of mitochondrial complexes (particularly Complex I) *via S*-nitrosylation and tyrosine nitration. This nitrosative stress can uncouple respiration, damage mitochondrial membranes, and ultimately lead to deleterious cellular consequences, highlighting a potential negative impact of excessive NO generation if antioxidant defenses are overwhelmed.^74^

**Fig. 5:**
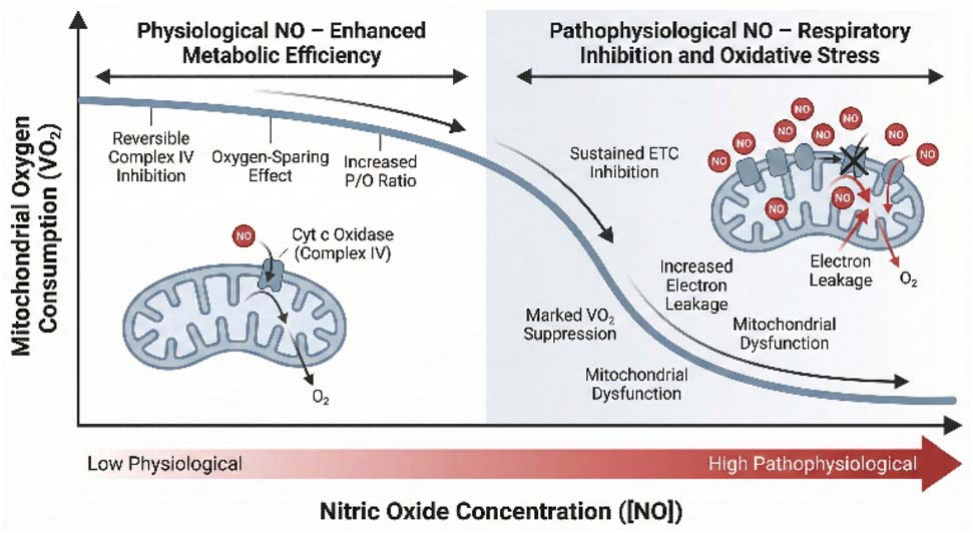
Biphasic effect of nitric oxide on mitochondrial respiration.

This improvement in mitochondrial efficiency is believed to be a primary mechanism underpinning the reduced oxygen cost of submaximal exercise observed after dietary nitrate supplementation (Fig. 5). The physiological consequence of this interaction is highly concentration-dependent. At physiological concentrations (typically in the low nanomolar range, ∼10–50 nM), NO acts to transiently and reversibly “throttle” mitochondrial respiration. This reversible competitive inhibition can be modeled mathematically using a competitive inhibition equation: *v* = V_max_}[O_2_]/K_m_(1 + [NO]/K_i_) + [O_2_], where the K_i_ for NO is extremely low (∼0.1–1 nM), making it a potent competitor against oxygen. Conversely, at pathophysiological concentrations (high nanomolar to micromolar range, >500 nM), NO—often *via* its conversion to peroxynitrite—causes irreversible inhibition of cellular respiration through covalent modifications such as *S*-nitrosylation and tyrosine nitration of the respiratory complexes. By making the mitochondria more efficient, the same amount of external work (*e.g.*, cycling at a fixed workload) can be performed with a lower rate of oxygen uptake. This would theoretically delay the onset of fatigue and enhance exercise tolerance. While some studies using isolated mitochondria have provided direct evidence for an improved P/O ratio after nitrate supplementation, other investigations have failed to replicate this finding, suggesting that the precise molecular mechanisms may be more complex or context-dependent.^75^

### 3.3. Interaction with calcium handling and muscle contractility

Emerging evidence suggests that NO generated from the nitrate–nitrite pathway can also influence muscle contractile function more directly by modulating calcium handling within the sarcoplasmic reticulum (SR). Studies in both human and rodent muscle have shown that nitrate supplementation can increase the expression of key calcium handling proteins, such as calsequestrin, and improve the efficiency of SR Ca^2+^ release and reuptake.^76^ These effects are likely mediated by cGMP-dependent signaling pathways or by direct *S*-nitrosation (a covalent attachment of an NO group to a thiol) of proteins like the ryanodine receptor (RyR1) and the SR Ca^2+^-ATPase (SERCA).^77^ By optimizing calcium cycling, NO may lead to a more forceful or efficient muscle contraction for a given level of activation, contributing to improvements in exercise performance, particularly during high-intensity, fatiguing tasks.

## 4. Impact on exercise performance

The translation of the chemical and physiological effects of the nitrate–nitrite–NO pathway into tangible improvements in human exercise performance has been the subject of intense investigation. While a large body of evidence supports an ergogenic role for dietary nitrate, the field is rife with conflicting findings, significant controversies, and a notable degree of unexplained variability in individual responses.

### 4.1. Evidence for ergogenic effects

The initial groundbreaking studies demonstrated that just a few days of dietary nitrate supplementation, typically in the form of beetroot juice, could reduce the steady-state oxygen uptake (VO_2_) during submaximal cycling and running by up to 5%.^78^ This reduction in the O_2_ cost of exercise was accompanied by a significant improvement in tolerance to high-intensity exercise, with subjects able to exercise for longer before exhaustion.^42^ Since these early reports, numerous studies have corroborated these findings across various exercise modalities. Meta-analyses have concluded that nitrate supplementation consistently improves time-to-exhaustion and time-trial performance, particularly in tasks lasting between 2 and 30 minutes.^42,79^ The benefits also appear to extend to high-intensity, intermittent exercise characteristic of team sports. For instance, Cosby and colleagues demonstrated that beetroot juice supplementation improved performance in a Yo–Yo intermittent recovery test, a common assessment of fitness in team-sport athletes. These effects are mechanistically linked to the physiological changes discussed previously: improved mitochondrial efficiency, enhanced muscle oxygenation, and potentially more efficient muscle contractility.^42,80^

To contextualize these mechanistic effects within human performance outcomes, Table 3 summarizes representative intervention trials linking nitrate supplementation protocols with exercise modalities and performance endpoints. A clear pattern emerges whereby reductions in submaximal VO_2_ and improvements in muscle efficiency translate more consistently into benefits for time-to-exhaustion, short-to-moderate duration time trials, and intermittent exercise, whereas elite endurance performance often shows null effects. This divergence likely reflects ceiling effects in highly trained athletes, baseline nitric oxide bioavailability, and limited scope for further enhancement of mitochondrial or contractile efficiency despite preserved biochemical responses.

**Table 3:** Summary of selected human trials on nitrate supplementation and exercise performance

Study	Population	Supplementation protocol	Exercise modality	Key findings	Conflicting evidence?
15	Recreationally active males	3 days NaNO_3_	Submaximal and maximal cycling	Reduced O_2_ cost of submaximal exercise; improved time-to-exhaustion	N/A
81	Recreationally active males	6 days beetroot juice	Cycling time trials (4 & 16.1 km)	Improved 4 km and 16.1 km time-trial performance	N/A
82	Trained cyclists	Acute beetroot juice	10 Km cycling time trial	No significant improvement in performance	Yes
83	Team-sport players	7 days beetroot juice	Yo–Yo intermittent recovery test	Improved intermittent sprint performance	Some studies show no benefit in repeated sprints^84^
85	Elite cyclists	6 days beetroot juice	30 min cycling time trial	No ergogenic effect observed	Yes
86	Recreationally active	7 days beetroot juice	Isometric and dynamic knee extension	Increased peak muscle power and torque	Some studies show no effect on maximal strength

### 4.2. Major controversies and discrepancies

Despite the positive findings, a significant number of well-controlled studies have reported no ergogenic benefit from nitrate supplementation, leading to major debates within the scientific community.

The influence of training status: one of the most prominent controversies revolves around the training status of the participants. The ergogenic effects of nitrate appear to be most pronounced in untrained or moderately trained individuals.^87^ In contrast, studies involving highly trained or elite endurance athletes have frequently failed to show performance improvements.^87,88^ Several compelling chemical and physiological reasons may explain this discrepancy.^89^ Elite athletes already possess highly optimized physiological systems as a result of chronic training. They typically exhibit higher baseline levels of plasma nitrate and nitrite and elevated eNOS expression and activity, leading to greater endogenous NO bioavailability.^90^ Their mitochondria are also more numerous and efficient, and they have enhanced muscle buffering capacity. Consequently, the relatively small physiological perturbations induced by dietary nitrate may be insufficient to elicit a further improvement in a system that is already near its biological ceiling.

**Fig. 6:**
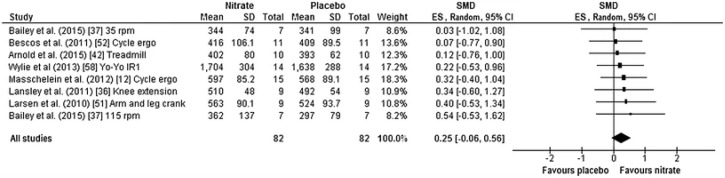
Forest plot of a meta-analysis on nitrate supplementation.

To contextualize the training-status controversy with quantitative evidence, Fig. 6 schematically summarizes a meta-analytic forest plot of nitrate supplementation effects on time-trial performance stratified by fitness level. The key visual message is not merely whether nitrate “works,” but how the distribution of effect sizes and their uncertainty shifts across subgroups: the pooled estimate in untrained/moderately trained participants is displaced toward performance improvement, whereas the elite subgroup clusters around trivial effects with confidence intervals that often overlap the null line. This pattern is consistent with the idea that training status acts as a moderating variable, increasing the likelihood that the true effect in elite cohorts is small, context-dependent, and difficult to resolve against day-to-day performance variability. Mechanistically, the subgroup divergence can be interpreted through a “diminishing returns” framework in which baseline nitric-oxide-related physiology constrains the marginal gain obtainable from exogenous nitrate. Studies indicate that endurance training enhances endothelial NO synthase signaling and NO-related biomarkers, and meta-analytic evidence suggests exercise training can elevate NO/nitrate–nitrite indices, implying a higher baseline NOx milieu in well-trained individuals.^91^ In such a setting, nitrate-induced increments in plasma nitrite (a proximate NO reservoir) may be proportionally smaller, and any downstream improvements in muscle oxygenation, mitochondrial efficiency, or contractile energetics may fall below the threshold required to translate into measurable time-trial gains. Importantly, Fig. 6 should also be read as a reminder that “no effect” in elite athletes is not equivalent to “no biology.” Rather, it may reflect a combination of (i) smaller true effects, (ii) heterogeneous responsiveness linked to baseline fitness and nitrite availability, and (iii) study-design constraints (event duration, dosing regimen, timing relative to competition, and control of oral microbiome). For example, aerobic fitness level has been shown to influence ergogenic responsiveness to nitrate, with different loading requirements proposed for elite *versus* lower-fit participants. Moreover, viewpoint syntheses emphasize that the current elite-athlete literature remains mixed and strongly conditional on experimental context, reinforcing why subgroup analyses are essential for a chemically grounded interpretation of the nitrate–nitrite–NO pathway in performance settings.^92^

Exercise modality and muscle fiber type: the benefits of nitrate may also be dependent on the type of exercise performed. The nitrate–nitrite–NO pathway is most active under hypoxic and acidic conditions. Therefore, it is logical that its effects would be most evident during high-intensity exercise that relies heavily on anaerobic metabolism and pushes muscle to its physiological limits.^89^ Furthermore, research suggests that nitrate supplementation may preferentially affect Type II (fast-twitch) muscle fibers. These fibers are recruited during intense exercise, have lower intrinsic blood flow and mitochondrial content, and are more susceptible to hypoxia than Type I fibers. Hernández *et al.* (2012)^93^ showed that nitrate supplementation selectively increased blood flow to fast-twitch muscle fibers in rats during exercise. This fiber-type-specific effect could explain why benefits are seen in sprinting and high-intensity intermittent tasks but may be less apparent in lower-intensity, prolonged endurance events dominated by Type I fiber recruitment.

### 4.3. Sources of inter-individual variability

A consistent observation in nitrate supplementation studies is the large variability in response between individuals, even within a homogenous group. Some individuals are clear “responders,” while others are “non-responders”.^94^ Understanding the sources of this variability is a key research priority.

**Fig. 7:**
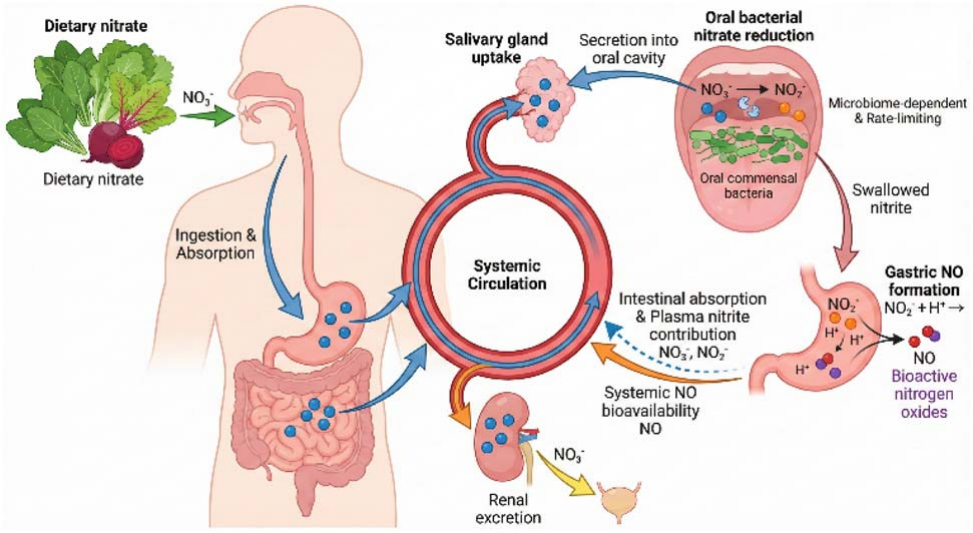
The entero-salivary circulation and microbial nitrate reduction.

The role of the microbiome: as the initial reduction of nitrate to nitrite is predominantly a microbial process, the composition and activity of an individual's oral microbiota are critical determinants of nitrate bioavailability (Fig. 7). There is substantial inter-individual variation in the abundance of nitrate-reducing bacteria on the tongue.^27^ Individuals with a microbial community rich in species like *Veillonella* may be more efficient at converting nitrate to nitrite and thus experience a greater physiological effect from supplementation.^95^ Lifestyle factors that alter the oral microbiome, such as diet, oral hygiene practices (*e.g.*, use of antiseptic mouthwash), and smoking, can therefore profoundly impact the efficacy of nitrate supplementation.

Genetic factors: genetic polymorphisms in genes related to NO signaling could also contribute to response variability. The gene encoding for endothelial nitric oxide synthase (*NOS3*) has several common polymorphisms that are known to influence enzyme activity and baseline NO levels.^96^ For example, individuals with certain *NOS3* genotypes may have naturally higher NO production, making them less responsive to an exogenous supply *via* the nitrate–nitrite pathway.^87^ While research into polymorphisms in genes for mammalian nitrate or nitrite reductases (like XOR) is still in its infancy, it is plausible that genetic variations affecting the function of these enzymes could also play a significant role. Collectively, these determinants of responsiveness do not act in isolation but converge on a limited number of biochemical control points within the nitrate–nitrite–NO pathway. As summarized in Table 4, training status, oral microbiome composition, genetic background, habitual diet, age, and muscle fiber distribution all modulate either baseline NO bioavailability or the efficiency with which exogenous nitrate is reduced to bioactive NO. For example, a high abundance of oral nitrate-reducing bacteria enhances nitrite delivery to the circulation, whereas genetic variants associated with elevated basal eNOS activity may reduce the relative contribution of the non-canonical pathway. Similarly, individuals with a greater proportion of type II fibers—characterized by lower oxygen tension and pH during exercise—provide a biochemical milieu that favors nitrite reduction to NO, amplifying functional effects at the muscle level. Importantly, these interacting factors help explain why identical supplementation protocols can yield divergent physiological outcomes despite comparable plasma nitrate exposure. Framing inter-individual variability through this multi-level lens underscores the need for phenotype-informed study designs and cautions against universal expectations of ergogenic benefit from dietary nitrate supplementation. Recent meta-analyses have begun to systematically quantify this inter-individual variability using meta-regression techniques. For example, meta-analytic data have confirmed a statistically significant inverse relationship between baseline aerobic fitness and the ergogenic effect size of nitrate supplementation,^97^ validating the “training status” hypothesis. While meta-analyses have robustly accounted for fitness level and exercise modality, quantitative meta-analytic data regarding the oral microbiome, genetic polymorphisms, and specific dietary interactions remain sparse, highlighting a critical area for future systematic reviews.

**Table 4:** Factors contributing to inter-individual variability in response to nitrate supplementation

Factor	Mechanism of influence	High responder profile (hypothesized)	Low responder profile (hypothesized)	References
Training status	Baseline NO bioavailability, mitochondrial efficiency, buffering capacity	Untrained or recreationally active	Highly trained elite athlete	87 and 89
Oral microbiome	Efficiency of nitrate-to-nitrite conversion in the entero-salivary circulation	High abundance of nitrate-reducing bacteria (*e.g.*, *Veillonella*)	Low abundance of nitrate-reducing bacteria; use of antiseptic mouthwash	27 and 95
Genetic polymorphisms	Baseline NOS activity, enzyme kinetics of reductases	Genotypes associated with lower basal eNOS activity	Genotypes associated with higher basal eNOS activity	96
Diet	Baseline nitrate intake, dietary factors influencing microbiome	Low habitual dietary nitrate intake	High habitual dietary nitrate intake (*e.g.*, vegetable-rich diet)	98
Age	Age-related decline in NOS function and vascular health	Older individuals with endothelial dysfunction	Young, healthy individuals with optimal NOS function	99
Muscle fiber type	Preferential effects on type II (fast-twitch) fibers	Individuals with a higher proportion of type II fibers	Individuals with a higher proportion of type I fibers	93 and 100

## 5. Methodological challenges in studying the pathway

Progress in understanding the nitrate–nitrite–NO pathway is often hampered by the significant technical challenges associated with accurately measuring NO and its metabolites in a dynamic biological system like exercising human muscle.^101^ The choice of analytical technique can profoundly influence experimental outcomes and their interpretation.^26^

### 5.1. Measurement of nitric oxide and its metabolites

Direct measurement of NO: nitric oxide is a highly reactive radical with a biological half-life of mere seconds, making its direct *in vivo* measurement exceedingly difficult.^102^ The most common method for real-time NO detection is the use of electrochemical sensors, such as porphyrin-coated microelectrodes.^103^ These sensors can be inserted into tissues to provide localized, real-time measurements. However, they face substantial limitations. A major challenge is selectivity; numerous other electroactive molecules present in biological fluids (*e.g.*, nitrite, ascorbate, dopamine) can interfere with the NO signal, leading to a poor signal-to-noise ratio.^104,105^ Furthermore, the sensor surface is prone to biofouling by proteins, which can degrade its sensitivity over time.^106^ While significant advancements in sensor design and coating technologies are ongoing, their application in contracting human muscle during dynamic exercise remains a formidable challenge.^105,107,108^

Indirect measurement *via* metabolites: due to the difficulties of direct NO measurement, most studies rely on measuring its more stable downstream oxidation products, nitrite (NO_2_^−^) and nitrate (NO_3_^−^), collectively termed NOx.^109–111^ These can be quantified in plasma, saliva, urine, and tissue biopsies using methods such as the Griess assay (a colorimetric method) or more sensitive techniques like chemiluminescence and high-performance liquid chromatography (HPLC). While valuable, using NOx as a surrogate for NO bioavailability has critical limitations.^104^ Plasma NOx reflects systemic NO production from all sources and may not accurately represent the localized, transient changes in NO concentration occurring within the microvasculature or mitochondria of the exercising muscle.^112^ Muscle biopsy samples provide a snapshot at a single time point but lack temporal resolution and can be affected by blood contamination.^113^ Therefore, while an increase in plasma nitrite following supplementation is a good indicator of substrate availability, it is an imperfect proxy for bioactive NO at the target site.

### 5.2. Comparing *in vivo* measurement techniques: microdialysis *vs.* sensors

To gain insight into the interstitial environment of muscle, researchers often turn to techniques like microdialysis. This involves inserting a thin probe with a semipermeable membrane into the muscle (*e.g.*, vastus lateralis).^114^ Small molecules, including nitrate and nitrite, diffuse from the interstitial fluid across the membrane into a perfusate, which is collected for later analysis.^115^ The primary advantage of microdialysis is its ability to sample a wide range of metabolites simultaneously. However, its major drawback is poor temporal resolution. There is a significant time lag associated with analyte diffusion and collection, making it unsuitable for capturing the rapid, second-by-second fluctuations in metabolites that occur during dynamic exercise.^116^

In contrast, electrochemical sensors offer superb temporal resolution, providing real-time data.^117^ However, as discussed, they are typically analyte-specific and suffer from interference and stability issues. A direct comparison highlights a trade-off: microdialysis provides a broader, time-averaged biochemical picture, while sensors provide a real-time but more narrowly focused and technically challenging view.^118,119^ The ideal approach, though technically complex, may involve the integration of both technologies to correlate real-time signals with comprehensive metabolite profiles.^120^ To systematize these methodological trade-offs, Table 5 summarizes the comparison of methodologies for measuring NO bioavailability in muscle. Importantly, the table highlights that techniques often grouped under “NO measurement” in fact interrogate distinct nodes of the nitrate–nitrite–NO continuum. Chemiluminescence and Griess-based assays quantify accumulated NOx pools, providing chemically robust but temporally integrated endpoints that are well suited for validating whole-tissue redox balance, yet insensitive to rapid exercise-induced dynamics. In contrast, electrochemical NO sensors probe transient, localized NO fluxes that more closely align with signaling events governing vasodilation and mitochondrial efficiency, albeit at the cost of selectivity and *in vivo* stability. Microdialysis occupies an intermediate position by directly sampling the interstitial compartment where nitrate reduction and nitrite-to-NO conversion occur, but its diffusion-limited recovery constrains kinetic interpretation. The inclusion of NIRS further underscores that functional readouts of oxygenation cannot be decoupled from biochemical NO measurements, reinforcing the need for multimodal strategies.

**Table 5:** Comparison of methodologies for measuring NO bioavailability in muscle

Methodology	Analyte(s)	Advantages	Limitations	References
Electrochemical sensors	NO (direct)	Real-time, high temporal resolution, localized measurement	Prone to interference (low selectivity), biofouling, technically challenging for *in vivo* human use	103 and 120
Chemiluminescence	NOx (nitrite/nitrate)	High sensitivity and specificity for NOx in fluids/homogenates	Indirect measure of NO, lacks spatial/temporal resolution, requires sample collection	115
Griess assay	Nitrite (indirectly NOx)	Simple, cost-effective, high-throughput	Lower sensitivity, interference from other compounds, only measures nitrite	121 and 122
Microdialysis	Nitrite, nitrate, other metabolites	Samples interstitial fluid directly, measures multiple analytes	Poor temporal resolution, invasive, recovery can be variable	114 and 123
Near-infrared spectroscopy (NIRS)	Deoxy-Hb/Mb, Oxy-Hb/Mb	Non-invasive, continuous monitoring of muscle oxygenation	Indirect measure of O_2_ supply/demand, not NO; limited tissue penetration depth	124 and 125

## 6. Conclusion and future directions

The nitrate–nitrite–NO pathway represents a fundamental pillar of mammalian redox biology and a crucial mechanism for regulating skeletal muscle physiology, particularly when the classical NOS pathway is constrained by low oxygen availability. The chemistry of this pathway is elegant in its simplicity and physiological relevance, transforming a common dietary anion into a potent signaling molecule precisely under conditions of high metabolic stress. The sequential reduction of nitrate to nitrite by microbial and mammalian enzymes, followed by the pH- and hypoxia-sensitive reduction of nitrite to NO by a host of deoxygenated heme proteins and mitochondrial enzymes, provides a robust system for matching NO bioavailability to cellular demand. This NO generation contributes to exercise hyperemia by promoting vasodilation and appears to enhance metabolic efficiency by modulating mitochondrial respiration, providing a strong mechanistic basis for the observed ergogenic effects of dietary nitrate supplementation.

Despite significant progress, critical gaps in our understanding remain, and the field is at a crucial juncture where a deeper focus on the underlying chemistry is required to resolve persistent controversies. Key future directions should include:

1. Quantifying chemical kinetics and thermodynamics: there is a pressing need for studies to determine the precise rate constants, activation energies, and thermodynamic parameters for the key nitrite reductase reactions (catalyzed by XOR, deoxy-Hb/Mb, CcO) within human skeletal muscle homogenates under physiologically relevant conditions of hypoxia and acidosis. This fundamental data is essential for building accurate computational models of NO flux during exercise.

2. Developing advanced *in vivo* sensing technologies: the limitations of current analytical methods severely restrict our ability to understand real-time NO dynamics. The development of next-generation wearable or minimally invasive biosensors with improved selectivity, stability, and signal-to-noise ratio for continuous monitoring of NO and nitrite in the muscle interstitium of athletes during competition is a paramount goal. Similarly, advancements in non-invasive imaging techniques to visualize NO dynamics in real-time would be transformative.

3. Integrating multi-omics approaches: to unravel the complex interplay of factors determining individual responses, future studies must integrate multi-omics approaches. Combining metagenomics (to characterize the oral/gut microbiome), genomics (to identify relevant polymorphisms in NOS and reductase genes), and metabolomics (to profile the broader metabolic response) will provide a holistic, systems-level understanding of the pathway and help pave the way for personalized nitrate supplementation strategies.

4. Conducting long-term longitudinal studies: most supplementation studies have been acute or short-term. Well-designed, long-term longitudinal studies are needed to assess the chronic effects of sustained nitrate intake on muscle adaptation, mitochondrial biogenesis, and performance, particularly in important but understudied populations such as the elderly, where it may help counteract age-related declines in vascular and muscle function.

In conclusion, the chemistry of the nitrate–nitrite–NO pathway provides a fascinating example of how the body harnesses simple inorganic chemistry to maintain physiological homeostasis under stress. While dietary nitrate is not a universal panacea for enhancing athletic performance, it is a powerful tool for modulating NO bioavailability with clear benefits in specific contexts. By addressing the current knowledge gaps through rigorous chemical, biochemical, and physiological investigation, we can better define its role and unlock its full potential not only in sports nutrition but also in clinical settings where tissue hypoxia and impaired NO signaling are prevalent.

## Author contributions

T. H. and Y. X. conceptualized the study and developed the methodology. T. H., L. L., and J. L. conducted the investigation, performed the literature search, and curated the data. T. H. and Z. Z. prepared the visualizations and figures. T. H. wrote the original draft. Z. Y. and Y. X. performed the formal analysis and contributed to writing – review and editing. Y. X. provided supervision and project administration. All authors have read and agreed to the published version of the manuscript.

## Conflicts of interest

There are no conflicts to declare.

## Data Availability

No primary research results, software or code have been included and no new data were generated or analysed as part of this review.
